# Parathyroidectomy Improves Survival In Patients with Severe Hyperparathyroidism: A Comparative Study

**DOI:** 10.1371/journal.pone.0068870

**Published:** 2013-08-05

**Authors:** Patricia Taschner Goldenstein, Rosilene Motta Elias, Lilian Pires de Freitas do Carmo, Fernanda Oliveira Coelho, Luciene Pereira Magalhães, Gisele Lins Antunes, Melani Ribeiro Custódio, Fábio Luiz de Menezes Montenegro, Silvia Maria Titan, Vanda Jorgetti, Rosa Maria Affonso Moysés

**Affiliations:** 1 Nephrology Division, Hospital das Clínicas, Faculdade de Medicina da Universidade de São Paulo, São Paulo, Brazil; 2 Head and Neck Surgery Division, Hospital das Clínicas, Faculdade de Medicina da Universidade de São Paulo, São Paulo, Brazil; University of Warwick – Medical School, United Kingdom

## Abstract

**Background and objectives:**

Secondary hyperparathyroidism (SHPT) in CKD is associated with an increased risk for mortality, but definitive data showing that parathormone control decreases mortality is still lacking. This study aimed to compare the mortality of patients with severe SHPT submitted to parathyroidectomy(PTX) with those who did not have access to surgery.

**Methods:**

This is a retrospective study in a cohort of 251 CKD patients with severe SHPT who were referred to a CKD-MBD Center for PTX from 2005 until 2012.

**Results:**

Most of our patients had indication of PTX, but only 49% of them had access to this surgical procedure. After a mean follow-up of 23 months, 72 patients had died. Non-survivors were older; more often had diabetes, lower serum 25 vitamin D and mostly had not been submitted to surgery. The relative risk of death was lower in the PTX patients (0.428; 95% CI, 0.28 to 0.67; p<0.0001). After adjustments, mortality risk was dependent on age (1.04; 95% CI, 1.01 to 1.07; p = 0.002), 25 vitamin D (0.43; 95% CI, 0.24 to 0.81; p = 0.006) and no access to PTX (4.13; 95% CI, 2.16 to 7.88; p<0.0001). Results remained the same in a second model using the PTX date as the study start date for the PTX group.

**Conclusions:**

Our data confirms the benefit of PTX on mortality in patients with severe SHPT. The high mortality encountered in our population is significant and urges the need to better treat these patients.

## Introduction

Mineral and bone metabolism disorders (CKD-MBD) are found almost universally in patients with chronic kidney disease (CKD) requiring dialysis. Secondary hyperparathyroidism (SHPT) is one of the most important and well-recognized of these disorders and data from observational studies have shown that CKD patients presenting high levels of serum parathormone (PTH) have an increased risk for mortality, as well as a higher morbidity [1], [2], [3].The Dialysis Outcomes and Practice Patterns Study (DOPPS) is the most important ongoing observational study and its results indicated that serum PTH higher than 600 pg/ml was associated with a 21% increase in all-cause mortality risk [3].

However, most of these observational studies compared patients with high levels of PTH with those presenting adequate levels of this hormone [4], [5] raising the question whether this mortality is related to SHPT itself or to other associated risk-factors. In addition, data that shows that controlling PTH in dialysis patients decreases mortality is still lacking [6], since most of the studies compared different strategies of therapy for SHPT, considering that not offering any therapeutic strategy would be considered as non-ethical.

Currently in Brazil, approximately 90,000 patients are being treated with dialysis, most of them reimbursed by the public health system [7]. Serum PTH is measured only two times per year and the current therapy for SHPT provided by this public system is calcium-based phosphate binders and calcitriol. Sevelamer is offered only to patients who present sustained hypercalcemia and paricalcitol or cinacalcet are not available yet. The consequence is that a significant proportion of our patients develop severe forms of SHPT and the only option for their therapy is surgical parathyroidectomy (PTX). However, few services in our country perform this surgery, leading to a waiting list in the reference centers, which is, at the biggest University in Latin America, of 563 days. This dramatic condition gave us the unique opportunity to test the hypothesis whether SHPT therapy, in this case through surgical PTX, is able to decrease mortality. To that end, we prospectively followed dialysis patients that were referred to our center for PTX and compared the mortality of those that were submitted to PTX with those who did not have access to this surgical therapy over time.

## Materials and Methods

**Figure 1:**
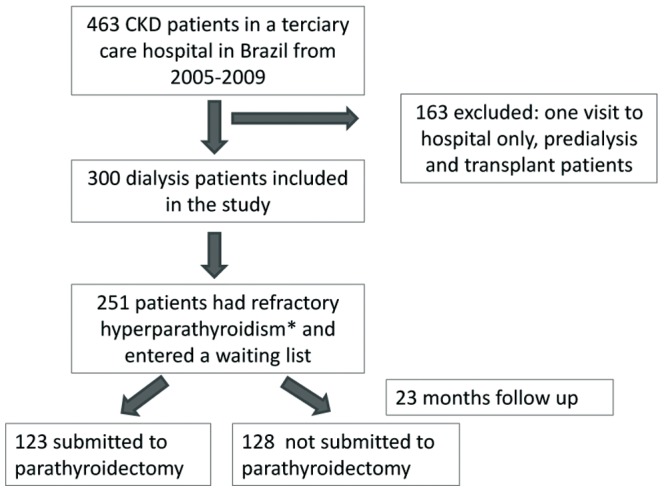
Flow chart of study design. Refractory hyperparathyroidism was defined as initial intact PTH greater than 800 pg/ml on calcitriol or in the presence of hyperphosphatemia and/or hypercalcemia, which prevented the use of calcitriol.

This historical analytical cohort encompassed CKD patients who attended the CKD-MBD clinic in a tertiary care hospital in São Paulo, Brazil (Hospital das Clínicas da Faculdade de Medicina da Universidade de São Paulo), from January 1, 2005 to December 31, 2009.This service is a reference center that receives patients from different dialysis services in São Paulo area for further evaluation and treatment. After excluding kidney transplant and predialysis patients, 300 individuals on dialysis were evaluated. Those patients that on initial evaluation had serum levels of intact PTH greater than 800 pg/ml on calcitriol or in the presence of hyperphosphatemia and/or hypercalcemia, which prevented the use of calcitriol, were considered as having refractory hyperparathyroidism and were referred to surgical PTX (n = 251). Patients selected for PTX were then entered in a waiting list according to the chronological order. A flow chart of the study design is shown in Figure 1. Even though the original criterion for surgery was time on the waiting list, the severity of symptoms (such as worsening of pain, tendon rupture, new fractures) was actually a main guide to anticipate surgery.

Total parathyroidectomy with autotransplantation in the forearm is the procedure of choice at our Institution. Intraoperative PTH levels were obtained before and after resection of each enlarged parathyroid gland with the goal to attain a final intraoperative PTH less than 100 pg/mL.

All patients of this cohort were followed until March 1, 2012, when a survival analysis was performed. Clinical assessments were performed on all patients. At baseline, serum PTH, total calcium, ionized calcium, phosphorus, alkaline phosphatase, vitamin D levels and medical record was queried.

This study was approved by the Ethics Committee of the Hospital das Clínicas da Universidade de São Paulo. As this was a retrospective study, our University Ethics Committee did not require an informed consent.

All data are expressed as mean ± SD for normally distributed data, median (min-max) for skewed data, and frequency (%) for categorical data. Comparisons between continuous variables and categorical variables were made using the t-test and chi-square test, respectively. The Kaplan-Meier method was used for the estimation of survival, and differences between the curves (PTX and non-PTX groups) were compared using the log-rank test. Data for survival analysis were censored at the time of renal transplantation or end of study (March 1^st^, 2012). Forward Stepwise (Likelihood Ratio) cox proportional hazards regression analysis was used to estimate the hazard ratio (HR) of non-PTX in the risk of death (variables in the model: sex, age, PTX, diabetes and 25 vitamin D). Lastly we performed a second analysis to exclude the “immortal time” bias, using the PTX date as the study start date for the PTX group. The same variables were entered on the adjusted models. Statistical analysis was performed using SPSS 17.0.1 (SPSS Inc, Chicago, Ill) and Graphpad prism 5 (CA, USA). A two-sided *P* value less than 0.05 was considered significant.

## Results

As shown in Table 1, patients that were referred to our center were relatively young, with a mean age of 47.9±13.5 years, with a slight predominance of men. We also had few diabetics, but more than 50% were hypertensive. Most had elevated ionized calcium and phosphate, as well as high alkaline phosphatase. Median PTH was high, whereas median 25 vitamin D was lower than normal [defined as ≥30 ng/ml (75 nmol/L)] and 22% of the patients were considered as having vitamin D deficiency [defined as <15 ng/ml (45 nmol/L), whereas vitamin D insufficiency was defined as serum 25 vitamin D levels between 15 and 30 ng/ml)]. Patients were symptomatic, as confirmed by the presence of continuous pain in more than 60% of them, although self-referred fracture was detected only in 13%. The only affordable therapy, calcitriol, was being used in 40% of the patients.

**Table 1:** Characteristics of the Patients at Baseline.

Variable		Reference Values
Male n (%)	163 (54.3)	-
Age (ys)	48 (13.5)	-
Diabetes n (%)	37 (12.6)	-
Hypertension n (%)	163 (58.6)	-
Ionized calcium (mg/dl)	5.1(3.7–7.1)	4.6–5.3 mg/dl
Phosphorus (mg/dl)	5.5 (1.62)	2.7–4.5 mg/dl
AP (U/L)	224 (46–3215)	40–129 U/L
PTH (pg/ml)	1.294 (10–7368)	150–300 pg/ml
25 vitamin D (ng/ml)# α	24 (3–97)	30–100 ng/ml
Dialysis vintage (months)	72 (3–288)	-
Pain n (%)	178 (63.7)	-
Fractures n (%)	37 (13.2)	-
Calcitriol Therapy (%)	112 (40.8)	-

Data expressed as mean (SD), median (min-max), or percentage. PTH = parathormone, AP = Alkaline phosphatase.

# 1 ng/mL = 2.5 nmol/L;

α data available for 218 patients.

Most of the patients (84.2%) had indication of PTX. When compared to those that did not need PTX, we could observe that these patients with refractory hyperparathyroidism presented higher serum phosphorus, alkaline phosphatase and serum PTH. They were also more symptomatic and had been on dialysis therapy during a longer period of time (Table 2).

**Table 2:** Comparison between groups of patients with indication versus no indication of PTX.

	Need of PTX (n = 251)	No need of PTX (n = 47)
Male gender n (%)	114 (45)	24 (51)
Age (ys)	47 (12.8)	51.2 (16.5)
Diabetes n (%)	27 (10.7)	9 (19.1)
Hypertension n (%)	139 (59.4)	24 (58.5)
Ionized calcium (mg/dl)	5.1 (3.7–7.1)	5.2 (4.2–6)
Phosphorus (mg/dl)	5.7 (1.6)	4.9 (1.5)*
AP (U/L)	262 (47–3215)	105.5 (46–522)*
PTH (pg/ml)	1457 (810–7368)	241 (10–794)*
25 vitamin D (ng/ml)# α	24(3–93)	27 (4–97)
Pain n (%)	159 (67)	17 (42.5) *
Fractures n (%)	33 (13.9)	4 (10)
On Calcitriol n (%)	92 (39.6)	19 (47.5)
Dialysis vintage (mo)	72 (3–288)	42 (3–168)*

* p<0.05 Data expressed as mean (SD), median (min-max), or number (percentage). PTH = parathormone. PXT = parathyroidectomy, AP = Alkaline phosphatase.

# 1 ng/mL = 2.5 nmol/L;

α data available for 218 patients.

After a mean follow-up of 23 months, only 123 out of the 251 patients with indication of PTX were submitted to this surgical procedure. We observed that 72 patients from this group of refractory hyperparathyroidism died [21 in the group that was submitted to PTX (7 deaths per 100 patient-year and 51 in the group that did not have access to the surgery (16 deaths per 100 patient-year); p<0.0001]. To better understand the factors that could be associated with an increased risk of death in this population of patients with severe hyperparathyroidism, we compared survivors and non-survivors, as shown in Table 3. The first group was younger, with a higher prevalence of males, and less frequently had diabetes. Ionized calcium, phosphate, alkaline phosphatase, as well as serum PTH were not able to differentiate groups. However, mean 25 vitamin D was lower in the non-survivor group, as well as the prevalence of patients that were submitted to PTX. We could also observe that the mortality rates were 19.4%, 30.7% and 47.5% in vitamin D replete, insufficient and deficient patients, respectively. However, a significant difference in terms of mortality was found only between the first and last groups of patients. Conversely, we could not find any beneficial effect of calcitriol on survival, since mortality rates were of 27.2% and 27.1% in patients that have been treated or not with this drug, respectively, at the time they were referred to our center.

**Table 3:** Characteristics of survivors versus non-survivors.

	Survivors (n = 179)	Non-Survivors (n = 72)
Male gender n (%)	88 (49.1)	26 (26)*
Age (ys)	44.6 (12.3)	53.9 (12)*
Diabetes n (%)	12 (6.7)	15 (20.8%)*
Hypertension n (%)	98 (57.9%)	41 (62.1)
Ionized calcium (mg/dl)	5.1 (3.7–7.1)	5.1 (4.3–6.5)
Phosphorus (mg/dl)	5.7 (1.5)	5.5 (1.6)
AP (U/L)	244 (69–2574)	296 (47–3215)
PTH (pg/ml)	1460 (810–7368)	1419 (811–5797)
25 vitamin D (ng/ml)# α	30.1 (SD 17.2)	(SD 10.9)*
Pain n (%)	110 (64.3)	48 (72.7)
Fractures n (%)	22 (12.8)	11 (16.6)
On Calcitriol n (%)	67 (39.6)	25 (39.6)
Parathyroidectomy n (%)	102 (56.9)	21 (29.1)*
Dialysis vintage (mo)	72 (3–264)	84 (8–288)

* p<0.0001 Data expressed as mean (SD), median (min-max), or percentages. PTH = parathormone, AP = Alkaline phosphatase.

# 1 ng/mL = 2.5 nmol/L;

α data available for 182 patients.

The comparison between patients submitted to PTX to those who did not have the chance to undergo surgery disclosed that the first group was younger and less frequently diabetic; however, they were more symptomatic, more often referring pain and fractures (Table 4). They also had higher serum calcium, phosphate, alkaline phosphatase and PTH and a longer dialysis vintage, turning them into more seriously ill patients. The analysis of the relative risk of death showed that it was lower in the PTX group than in the non-PTX group (0.428; 95% confidence interval, 0.28 to 0.67; p<0.0001).

**Table 4:** Characteristics of patients submitted versus not submitted to PTX.

	Submitted to PTX (n = 123)	Not submitted to PTX (n = 128)
Male gender n (%)	57 (46.3%)	57 (44.5%)
Age (ys)	46 (37–54)	50 (40–58)*
Diabetes n (%)	7 (5.6%)	20 (15.6%)*
Hypertension n (%)	62 (50.4%)	77 (60.1%)
Ionized calcium (mg/dl)	5.2 (4.95–5.5)	5 (4.8–5.3)*
Total calcium (mg/dl)	10 (9.37–10.63)	9.7 (9.22–10.1)*
Phosphorus (mg/dl)	5.7 (5–6.9)	5.3 (4.2–6.6)*
AP (UI/L)	335 (161.5–559)	246 (135–441)*
PTH (pg/dl)	1554 (1168–2300)	1360 (1026–1967)*
25 vitamin D (ng/ml)# α	23.5 (15.25–38.5)	24 (16–32.5)
Pain n (%)	82 (66.6%)	77 (60.1%)
Fractures n (%)	21 (17%)	12 (9.3%)
On Calcitriol n (%)	40 (32.5%)	52 (40.6%)
Dialysis vintage (mo)	84 (48–120)	72 (36–120)*

* p<0.05 ; Data expressed as mean (SD), median (min-max), or percentages. PTH = parathormone; PTX = parathyroidectomy, AP (Alkaline Phosphatase).

# 1 ng/mL = 2.5 nmol/L;

α data available for 182 patients.

**Figure 2:**
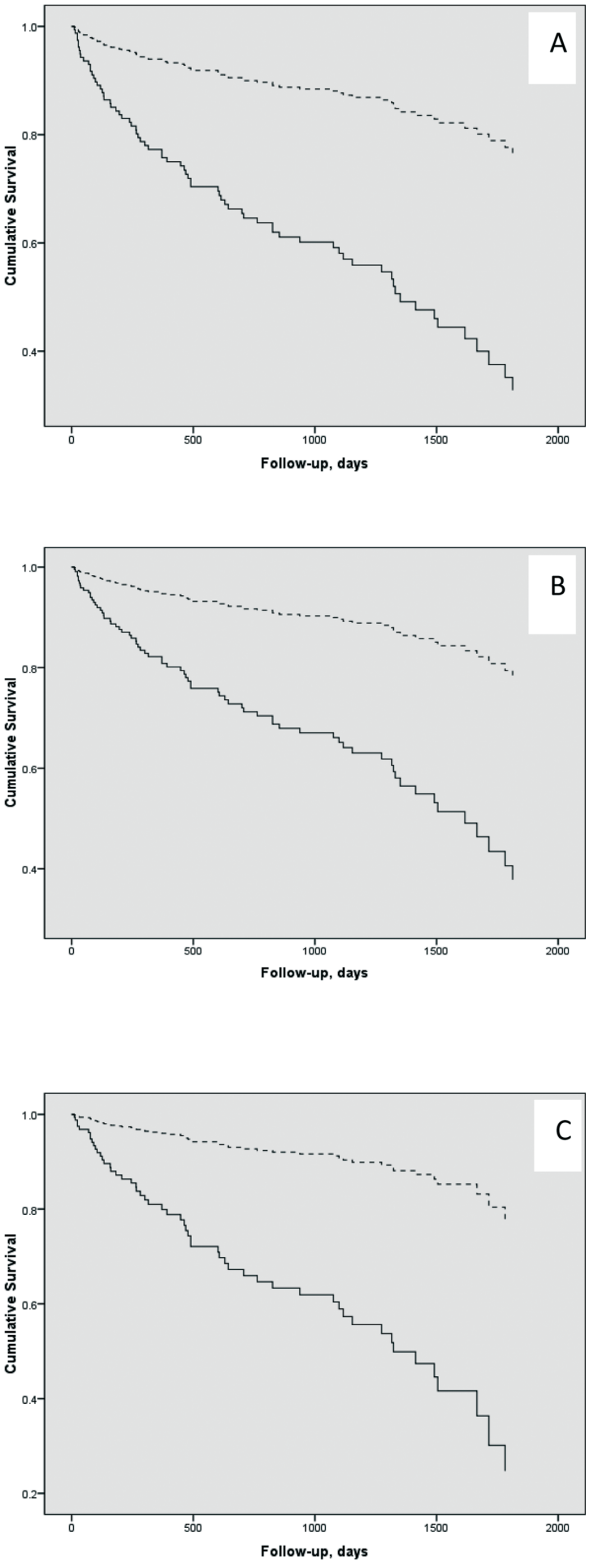
Kaplan-Meier survival curves according to PTX. A higher median survival time was observed for PTX group (2185 days) [superior dashed line] than non-PTX group (1357 days) [continuous line]. Log-rank p<0.0001 for curves A (unadjusted), B (adjusted for age, gender and diabetes) and C (adjusted for age, gender, diabetes and vitamin D).

**Figure 3:**
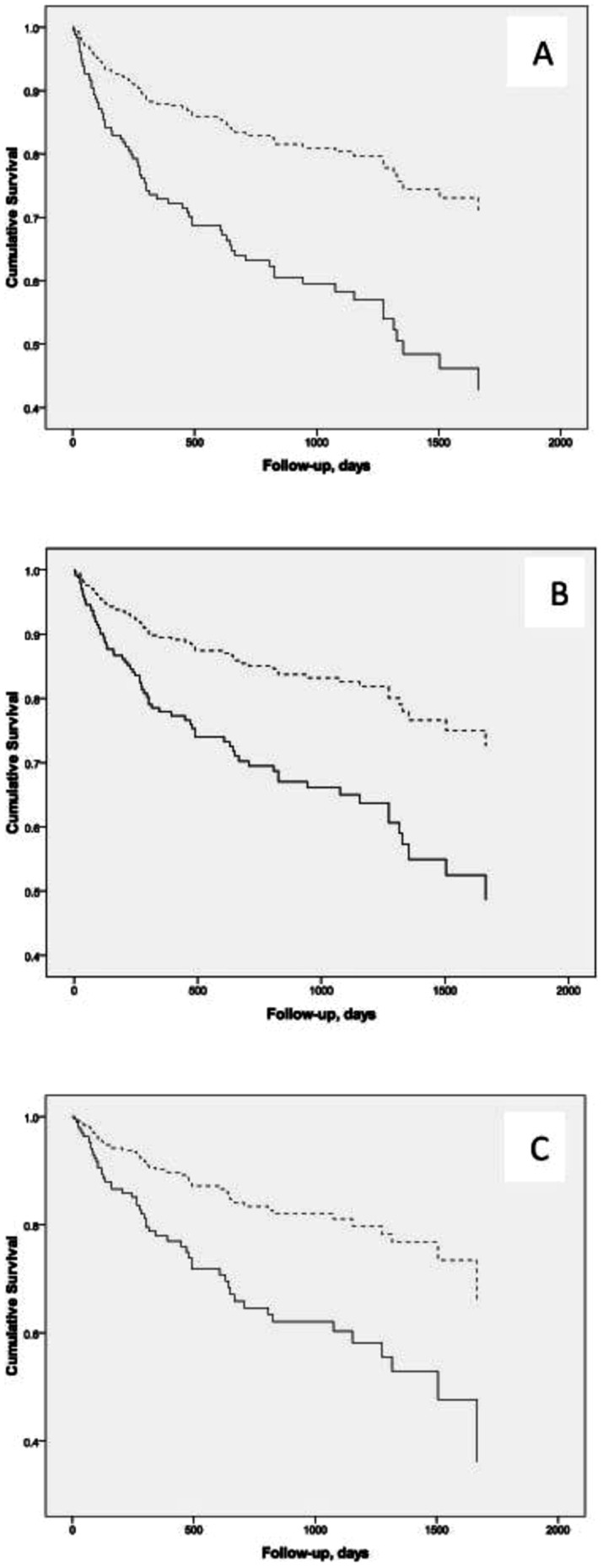
Kaplan-Meier survival curves according to PTX in the second model, using the PTX date as the study start date for the PTX group. A higher median survival time was observed for PTX group [superior dashed line] than non-PTX group [continuous line]. Log-rank p<0.0001 for curves A (unadjusted), B (adjusted for age, gender and diabetes) and C (adjusted for age, gender, diabetes and vitamin D).

Survival curve showed a benefit of PTX, as depicted in Figure 2 (log-rank test p<0.0001). In unadjusted model the relative hazard in the non-PTX group vs. the PTX group was 4.13; 95% confidence interval [CI], 2.46 to 6.92; p = 0.0001. After adjustment for age, gender and presence of DM, mortality risk was dependent on age (1.06; 95% CI, 1.04 to 1.08; p<0.0001) and no access to PTX (3.91; 95% CI, 2.30 to 6.66; p<0.0001). After adjustment for age, gender, DM and 25 vitamin D, the variables that remained significant were age (1.04; 95% CI, 1.01 to 1.07; p = 0.002), 25 vitamin D (0.43; 95% CI, 0.24 to 0.81; p = 0.006) and no-PTX (4.13; 95% CI, 2.16 to 7.88; p<0.0001). Finally, we performed another multivariable analysis, in order to rule out the “immortal time” bias, and we observed that the relative hazard in the non-PTX group vs. the PTX group was 2.5, 2.2 and 2.4 using the same models described before (Figure 3). Therefore, the benefits of PTX were still confirmed in this unique population with severe form of hyperparathyroidism.

## Discussion

Secondary hyperparathyroidism (SHPT) is a common complication in patients on chronic hemodialysis. Conventional medical therapy with first generation of vitamin D receptor activators (VDRAs), such as calcitriol and alfacalcidol, lack adequate long-term control of intact parathyroid hormone (iPTH) in a large group of patients, resulting in vascular calcification [8] and an increase of cardiovascular mortality [9].

The exact mechanisms by which altered mineral metabolism increases mortality and morbidity remain unclear, but vascular calcification has been proposed as a possible link [8], [10]. It has been described that serum phosphorus stimulates the transformation of vascular smooth muscle cells into osteoblast-like cells, creating an environment susceptible to calcification [11], [12]. Similarly, hypercalcemia and uremia cause vascular calcification by active processes suggesting the importance of strict management of uremia, serum minerals and PTH in dialysis patients [8], [13], [14].

Parathyroidectomy is the treatment of choice for patients who become resistant to medical therapy. A small clinical study has shown slowing of calcification progression after PTX [15], but there has been a few reports of improved survival post-PTX [16], [17], [18]. This surgical procedure seems to improve high blood pressure, anemia, nutritional state, immunity, glucose and lipid metabolism, muscle strength and quality of life, relieves insomnia and even increases cognitive function [19], [20], [21], [22], [23].

The mechanisms responsible for these remarkable beneficial effects of PTX are not certain; however, the dramatic reductions in PTH and better control of serum minerals are certainly involved. Iwamoto et al [4] performed a retrospective comparison of survival and mineral metabolism between 88 patients who had undergone PTX and 88 control dialysis patients with no indication of PTX. The overall survival rate was 90.4% in the PTX group and 67.4% in the control group, whereas the cardiovascular death-free survival rate was significantly better in the PTX group (94.6% versus 76.3%). They found that besides reducing PTH, calcium levels were significantly lower in the PTX group compared to the control group and suggested that these efects could contribute to the decrease of cardiac afterload and inhibition of the further progression of vascular calcification resulting in a better prognosis of this group of patients. Sharma et al also have described an overall improvement in mortality from cardiovascular diseases in 86 patients who had this time undergone near total parathyroidectomy compared to matched controls [5]. However, both of them performed retrospective studies and their control group was composed of dialysis patients with no indication of PTX, which makes the groups distinct, and the presence of other confounding factors contributing to this benefit of PTX could not be ruled out.

In our center, although more than two surgeries were performed each month, the number of patients referred to us and the mean time on waiting list were astoundingly elevated. As a consequence, sometimes we had to anticipate surgery in those patients that were more symptomatic and severely ill. The final result is that the group that was submitted to PTX was younger, but with a more severe manifestation of SHPT. Nevertheless, the adjusted survival curve was able to confirm the benefit of therapy in this group of patients that currently is rarely seen in the US, Japan or Western Europe.

During the follow up, we found a mortality rate of 29% in the group patients with refractory hyperparathyroidism, which was even higher in the group of patients who needed, but was not submitted to PTX (40%). The median non-PTX patients' follow-up time was shorter than PTX patients' follow-up time (493 vs. 763 days; p = 0.15), but did not reach statistical significance. If we considered the PTX date as the study start date for the PTX group, the results basically remained the same, although the HR of mortality for non-PTX group had diminished from 4.0 (as previously showed) to 2.4 (unadjusted) and 2.1 (adjusted for age, gender and presence of diabetes).

Kestenbaum et al [24] have demonstrated that long-term relative risks of death among patients undergoing PTX were estimated to be 10% to 15% lower than those of matched control patients not undergoing surgery. Median survival was higher in the PTX group than in matched control group. Although these authors also found a benefit of PTX, this study differs from ours in several aspects: first, the control group did not have indication for PTX; second, the patients had full access to medication; third, the PTX was not delayed due to extensive waiting list; finally, the above mentioned study compared patients and controls from many centers, which may have some difference in clinical approach, as well as indication of PTX. The more pronounced benefit of PTX in our study is probably related to the differences mentioned above.

When we compared survivors and non-survivors we could observe that survivors were younger and mostly males with less frequent diabetes. Interestingly, neither serum minerals not even PTH was able to differentiate groups. On the other hand, parathyroidectomy and mean levels of 25 vitamin D were crucial to differentiate the group of survivors versus non-survivors. This association of serum 25 vitamin D and survival has already been shown in other studies [25], [26]. In our study, we could observe a progressive increase in mortality rate as the serum levels of 25 vitamin D decreased. However, a significant difference in terms of mortality was found only between vitamin D replete in comparison to vitamin D deficient patients. Curiously, after the initial evaluation, all patients with hypovitaminosis D were prescribed with cholecalciferol, what would normalize their vitamin D status, at least in theory. Another interesting finding is related to the absence of any significant effect of calcitriol administration on mortality. Previous studies have shown that active vitamin D administration decreases mortality in dialysis patients with hypovitaminosis D [25]. However, in our sample, mortality rates were similar between those treated or not with calcitriol. One possible explanation is that those non-classical benefits of calcitriol therapy could not be observed in these patients with a severe form of SHPT.

In the literature, there is still an intense debate on the better strategy for SHPT management. Some authors believe that it should start with VDRAs [27], whereas others prefer calcimimetics [28]. A recent published study, which compared the effects of calcimimetics with conventional therapy with VDRAs, showed inconclusive results [29]. Curiouslly, one possible explanation for the EVOLVE disappointing results was the high rate of PTX in the control group, which might have decreased the number of events in those patients, i.e. the effect of cinacalcet could be masked by that imbalance in PTx between groups. A dangerous conclusion that might be drawn from the EVOLVE results [6] is to whether there is any benefit of cinacalcet or any SHPT therapy in terms of mortality and morbidity. Our data, generated from a critical situation in the Brazilian health system comes to confirm that not giving any therapeutic option to these patients increases significantly their risk of death. In Brazil and other developing countries, dialysis is affordable for most of the CKD patients and this could give them a chance to a better survival. However, if this access to dialysis does not come with medical therapy for CKD-MBD and other CKD complications, our patients will still be exposed to a high risk of mortality, as well as of fractures and pain. One piece of information that could not be given by our data is whether surgical PTX is better than current conservative therapy, which includes new generations of VDRAs and calcimimetics, since any of our patients have the opportunitty to be treated with these drugs.

This work has several limitations. The number of patients is relatively small; this is a cohort study, in which patients were not randomized to treatment with PTX or not, and the more seriously ill were eventually submitted to surgery before. Like other observational studies, the present data is also prone to selection bias, implying the risk of residual confounding. Additional limitations are some unmeasured patient characteristics, such as serum 25 vitamin D and presence of undetected clinical comorbidities, which limit the adjustments and inferences that can be made. Notwithstanding, we had the unique opportunity to have a control group of real life with indication, but no therapy to their disease, which would not be accepted in a classical clinical study. We believe that the high mortality encountered in our population is significant and urges the need to treat these patients.

## Conclusion

The definitive information that we can provide is that, although being seriously ill, symptomatic, with high serum phosphorus, very elevated PTH and low vitamin D, in an extreme situation, our patients were clearly benefited from PTX.
